# Metal-Dielectric Nanopillar Antenna-Resonators for Efficient Collected Photon Rate from Silicon Carbide Color Centers

**DOI:** 10.3390/nano13010195

**Published:** 2023-01-01

**Authors:** Faraz Ahmed Inam, Stefania Castelletto

**Affiliations:** 1 Department of Physics, Aligarh Muslim University, Aligarh 20002, India; 2 School of Engineering, RMIT University, Melbourne, VIC 3001, Australia

**Keywords:** emission electromagnetic-dynamics, Mie scattering, nanopillars, silicon carbide, vacancy silicon, single-photon sources, fluorescence collection enhancement

## Abstract

A yet unresolved challenge in developing quantum technologies based on color centres in high refractive index semiconductors is the efficient fluorescence enhancement of point defects in bulk materials. Optical resonators and antennas have been designed to provide directional emission, spontaneous emission rate enhancement and collection efficiency enhancement at the same time. While collection efficiency enhancement can be achieved by individual nanopillars or nanowires, fluorescent emission enhancement is achieved using nanoresonators or nanoantennas. In this work, we optimise the design of a metal-dielectric nanopillar-based antenna/resonator fabricated in a silicon carbide (SiC) substrate with integrated quantum emitters. Here we consider various color centres known in SiC such as silicon mono-vacancy and the carbon antisite vacancy pair, that show single photon emission and quantum sensing functionalities with optical electron spin read-out, respectively. We model the dipole emission fluorescence rate of these color centres into the metal-dielectric nanopillar hybrid antenna resonator using multi-polar electromagnetic scattering resonances and near-field plasmonic field enhancement and confinement. We calculate the fluorescence collected photon rate enhancement for these solid state vacancy-centers in SiC in these metal-dielectric nanopillar resonators, showing a trade-off effect between the collection efficiency and radiative Purcell factor enhancement. We obtained a collected photon rate enhancement from a silicon monovacancy vacancy center embedded in an optimised hybrid antenna-resonator two orders of magnitude larger compared to the case of the color centres in bulk material.

## 1. Introduction

A key problem for many of the applications for example of the nitrogen vacancy (NV) centres in diamond [1] and of the more recently studied vacancy related or intrinsic color centres in silicon carbide (SiC) [2,3], is the overall collected photon rate, CPR, as this is linked to the readout fidelity of their electron spins, thus impacting these color centres application in quantum sensing and as spin-photon interface system for entanglement distribution.

The collected photon rate, CPR = $F_{p}\cdot CE$ [4,5,6], is proportional to the modified Purcell factor, $F_{p}=\frac{\gamma }{\gamma_{bulk}}$, where γ values are the corresponding decay rates in the nanostructure and in the bulk material, and the photon collection efficiency (CE), which depends on the spatial overlap of the emission with the collection optics.

Nano and micro-pillars have been fabricated in the last decade for improving the CE of single-photon emission for point defects integrated inside them. However, these nanostructures are designed to mainly act as a vertical waveguide to improve the emission spatial overlap with the collection optics, namely with a high numerical aperture (NA) microscope objective out of the sample plane. Thus only the CE along the scanning confocal microscope objective is generally enhanced. A moderate F${}_{p}$ factor of ≈ 2 can be achieved, for optimised nanopillars size with respect to their height to diameter ratio and positioning of the emitter in the nanopillar vertical extension [7].

In particular nanopillars in diamond and in SiC have been fabricated to enhance their color centres emission collection [8,9,10,11,12,13,14,15,16], and have been proposed to improve imaging and quantum sensing of magnetic field [13,17] and nanothermometry [18]. For color centre with optical spin read-out, the sensitivity of the measurement of magnetic field [19] is $\propto \frac{1}{g_{e}\cdot C\sqrt{CPR\cdot n_{spin}\cdot T_{2}}}$, where $g_{e}$ is the spin defect gyromagnetic ratio, *C* is the optical spin read-out contrast, $n_{spin}$ are the number of spins in the probed volume and $T_{2}$ is the spin-echo coherence time under the specific sensing sequence. As such, optimising CPR is key for quantum sensing applications with ensemble of spin defects in diamond and SiC.

We have recently modelled the optimal height to diameter ratio and the location of SiC single emitter based on the nitrogen vacancy centre, $N_{C}V_{\mathrm{Si}}$ [12,20,21,22], which has a room temperature spectrum centered at 1250 nm, within a single nanopillar [7] to improve this specific color centre fluorescent enhancement.

The experimentally measured fluorescence enhancement rate, FE, is defined by the equation [12,23,24]:

(1) $$FE=F_{p}\cdot \frac{CE}{CE_{bulk}}\cdot \frac{QE}{QE_{bulk}},$$

$QE_{bulk}$, and QE are the color center’s bulk and in the nanopillars quantum efficiency assumed to be the same. CE and $CE_{bulk}$ are the collection efficiency (CE) in SiC nanopillars and in the bulk, whose ratio represents the CE enhancement. The CE for a vertical dipole in SiC bulk was determined to be 0.45%. Most of the experimental work so far has attributed the FE to the increased CE in the nanopillars with generally values of experimental fluorescence enhancement in the range 4–16 [10,11,12,13,14,15].

By modelling, the optimum SiC cylinder’s height was found to be around 1600 nm in the case of emission centred at 1300 nm, corresponding to the N${}_{C}V_{Si}$ in SiC [22] with the optimum diameter being 560 nm (somewhat less than λ/2). The optimal position of the emitter within the nanopillars is also at λ/2 = 650 nm from the top of the nanopillar. For optimal conditions the $F_{p}\approx$ 2.3 and CE = 40%, thus a CPR = 0.92. Here the emitter dipole is considered along the c-axis of the nanopillars. However for most of the c-axis oriented N${}_{C}V_{Si}$ centers, both the excitation and the emission degrees of polarisation are larger than 90% suggesting a dipole orientation not along the c-axis [22]. This result is due to the assumption of a vertical dipole emission coupling with the vertical Mie resonance modes of the nanopillars responsible for an enhancement of the local density of states (LDOS) for the embedded dipole within the cylinder.

For an horizontal dipole (as also shown in the following) this effect is not present. While the CE can be higher than 45%, it occurs for a lower pillar radius (400 nm) from where the relative decay rate is maximum (560 nm). The dipole emission is therefore not coupled to the upward propagating mode of the cylinder and the CPR = 0.33.

We have also studied the FE of the $N_{C}V_{\mathrm{Si}}$ center in an hybrid dielectric-plasmonic nanostructure made of a SiC nanopillar with the fluorescence collected by a diameter matched cylindrical Ag nanoantenna on top of the nanopillar [25].

Specifically, for an optimised pillar of h${}_{2}$ = 720 nm height and diameter D = 560 nm matched with the Ag nanoantenna with same diameter and height of h${}_{1}$ = 990 nm, the CE = 24% and F${}_{p}$ = 7–8 for a vertical dipole associated with $N_{C}V_{\mathrm{Si}}$ very close (d ≈ 20 nm) to the Ag nanoantenna, providing a CPR = 1.68 and a FE = 700. If the dipole is d ≈ 300 nm from the antenna the CE = 55% however the F${}_{p}$ = 2, obtaining a CPR = 1.1.

**Figure 1:**
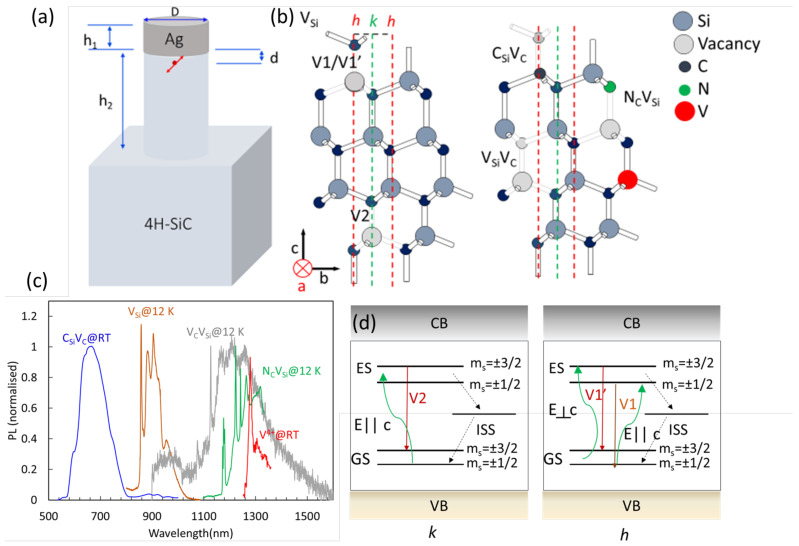
(**a**) Nanopillar antenna-resonator in 4H-SiC and Ag, where optimization of the nanopillar and antenna height (h${}_{1,2}$), nanopillars diameter (D), dipole distance from the antenna (d) are studied. (**b**) Crystallographic representation of 4H-SiC with hexagonal (h) and cubic (k) sites location for Si and C, showing the h and k sites for the V${}_{\mathrm{Si}}$ and the h site for carbon vacancy pair ($C_{\mathrm{Si}}V_{C}$) and $N_{C}V_{\mathrm{Si}}$, and the k site for the divacancy $V_{C}V_{\mathrm{Si}}$ and Vanadium (V). The direction of the c-axis is also shown. (**c**) Spectroscopy at room and low temperature of here studied color centres. Single $C_{\mathrm{Si}}V_{C}$ at room temperature (RT) measured using a home-built scanning confocal microscope with 532 nm excitation as described in ref. [27] based on samples fabricated as described in ref. [28]. Ensemble of V${}_{\mathrm{Si}}$ implanted on a c-face grown 4H-SiC, showing the ZPLs of the V1/V1’ lines for the centre at the h site and the V2 line for the centres at the k site. As the substrate is grown on the c-face the dominant emission is the V1’ due to its dipole laying in the plane of the substrate (excited by a laser perpendicular to the c-axis). Additional PL at 12 K of ensemble of $V_{\mathrm{Si}}V_{C}$, $N_{\mathrm{Si}}V_{C}$ and RT $V^{+4}$. $V_{\mathrm{Si}}$, $V_{\mathrm{Si}}V_{C}$, and $N_{\mathrm{Si}}V_{C}$ are excited using 780 nm and 976 nm respectively and measured using a commercial Horiba Raman system and home built confocal systems operating at low and room temperature equipped with infrared spectrometer and single photon detectors as described in ref. [12,27]. Samples are created as described in ref [12]. $V^{+4}$ was not measured but deduced from ref. [29]. (**d**) Schematic of the energy levels (Ground state, GS, excited state, ES, and intersystem state, ISS) of the V1’/V1 and V2 emitters, showing that V1 and V2 are excited with an electric field parallel to the c-axis, while V1’ with an electric field perpendicular to the c-axis.

In the present work we study the CE, F${}_{p}$ and CPR in a nanopillar-resonators antenna in the geometry shown in Figure 1a, with embeddings of the other relevant known color centres in SiC such as the most prominent silicon monovacancy centre in SiC, $V_{\mathrm{Si}}$ [26], as well other centres such as the carbon antisite vacancy pair $C_{\mathrm{Si}}V_{C}$, the divacancy $V_{\mathrm{Si}}V_{C}$ in 4H-SiC (Figure 1b–d). Compared to the previous work, here we focus on optimising the CPR values for more realistic experimental conditions, including considering the so-far-observed optical properties of these color centre.

Specifically we consider the observed most typical dipoles polarisation and other emission characteristics such as the Debye–Waller (DW) factor in evaluating the CPR. The aim of studying various color centres within this hybrid dielectric–plasmonic nanostructure is to determine realistic design parameters with feasible fabrication for enhanced CPR. In this system, multi-polar electromagnetic scattering resonances and near-field plasmonic field enhancement and confinement are combined. The Ag cylinder acts as a resonator and antenna for a dipole radiating close to the Ag surface, providing higher values of F${}_{p}$ and quality factor. The Ag is an efficient antenna for a radiating dipole relatively far from the Ag surface directing the dipole’s power effectively into the far-field free space. In this configuration it is achieving higher CE than high Purcell enhancement. CPR optimisation is a trade off between CE and F${}_{p}$ enhancement.

## 2. Color Centres Collected Photon Rate

In the following we briefly describe the relevant known properties of these colour centres in SiC and their collected photon rate (CPR) based on these properties.

The photo-luminescence spectra of these color centres is relevant to determine the CPR, and they are shown in Figure 1c with the zero-phonon lines (ZPLs) when the spectra are at low temperature, while generally a large phonon side band is present at room temperature in their emission, making the CPR enhancement challenging.

4H-SiC is a compound of Si and C and has a hexagonal lattice with two non-equivalent lattice sites known as the hexagonal site, h, and the quasi-cubic site, k. These two sites allow for four defect orientations (hh, hk, kh, and kk) for the defects occupying two sites such as, for example, the double vacancy of carbon and silicon. Each defect orientation corresponds to a specific ZPL and as well dipole orientation. In general axial orientations of the defects (hh, kk) are associated to defects dipole mostly orthogonal to the c-axis, while basal orientations (kh and kk) a mixture of dipole polarisations can occur. Here we briefly describe the properties of the defects assumed in the nanopillars antenna-resonators and refer for more details on their properties to some recent overview [30].

The monovacancy of Si, $V_{\mathrm{Si}}$, in 4H-SiC has two non equivalent crystal sites (cubic, k, and hexagonal h), giving rise to two ZPLs at 862 nm (V1, h site) and 917 nm (V2, k site). Its spin number is 3/2. V1’ (858.2 nm) [31] corresponds to the higher energy excited state of the ZPL at the h site locations (see Figure 1d). Both V2 and V1 (V1’) lines have been studied for optical coherent spin control and qubit [32,33], single photon source [34] spin photon interface [35] and a source of indistiguishable single photon sources [36,37] and magnetic field sensing [38]. For SiC grown on the c-plane (0001), the V1’ line is polarized with the laser field (dipole) perpendicular to the c-axis, while the V1 and V2 lines with the laser field (dipole) parallel to the c-axis. Thus both the V1 and V2 ZPLs possess a dipole orientation along the vertical direction (perpendicular) to the bulk substrate [10], with an emitter field propagating in plane, so amenable for in-plane photonics. By growing epitaxially a SiC layer on a-plane (rather than the c-plane), it is possible to have the V1 and V2 lines dipoles fully in plane. As such the $V_{\mathrm{Si}}$ allows to have perfectly in plane and vertical dipoles as opposite to NV in diamond whose dipole orientation is around $35^{\circ }$ from the vertical direction [14]. This makes the here considered color centre amenable for in-plane nanophotonics or for nanopillar fluorescence enhancement that is particularly needed for vertical dipole orientation, and thus out-of plane photonics.

Divacancies ($V_{\mathrm{Si}}V_{C}$) [39,40] in 4H-SiC has been identified in the four configurations of the lattice sites with associated different ZPLs known as PL1-4. The defect oriented along the c-axis *(kk)* 1131 nm (PL2) and *(hh)* 1132 nm, are mainly excited by the electric field perpendicular to the c-axis (E⊥c), while basal emitters have ZPLs 1108 nm, 1078 nm with mixed excitation polarisation. Their emission, even if it is not in the telecom band, is still relevant for fiber communication if compared to the NV centre in diamond.

The $V_{\mathrm{Si}}V_{C}$ in the *(hh)* and *(kk)* due to its $C_{3v}$ symmetry, possess spin excited and ground state fine structures permitting optical spin polarisation at $m_{s}=0$ and consequent possible spin maninpulation and optical read-out. The $V_{\mathrm{Si}}V_{C}$ are one of the main candidate for single-shot spin readout and spin-photon interfacing experiments using a photonic cavity with a record coherence time of 5 s [41]. High-contrast readout and coherent manipulation of a single $V_{\mathrm{Si}}V_{C}$ electron spin at room temperature was recently achieved [42] for divacancies within stacking fault (s.f.) locations acting as quantum wells (associated to PL5 and PL6 at 1042 nm and 1038 nm). Their excitation polarisation has been determined to be orthogonal to the c-axis (E⊥c) for PL5 and parallel (E‖c) for PL6 [43]. These type of divacancies are an ideal candidate for magnetic sensing at ambient conditions.

The positively charged state $C_{\mathrm{Si}}V_{C}$ in 4H-SiC has shown a bright room-temperature photon emission at ≈ 650–670 nm wavelength [28]. In ensemble it possess eight ZPL known as AB lines due to four not equivalent sites with two excited states. Some of the AB lines are excited with electric field parallel and perpendicular to the c-axis (axial defects) or both (basal defects) [28]. Its spin number S=1/2, while ODMR has been observed yet. Vanadium (V${}^{4+}$) dopant is replacing a silicon atom and is an appealing impurity since its ZPLs are directly in the telecom O-band, with an excited state lifetime of the order of tens of nanoseconds, and a S=1/2 electronic spin due to a single unpaired electron. Single vanadium centres have been isolated, and optically-detected magnetic resonance has been demonstrated using all optical pump and probe [44].

The most important properties of these color centres that underpin their application in quantum technologies, specifically spin-photon interface are reported in Table 1 including the electric field excitation polarisation, ZPLs, the optical lifetime $\tau_{fl}$, the Debye–Waller (DW) factor χ (the proportion of radiative emission occurring through the zero-phonon transition compared to the transitions to all vibronic levels), the optical spin-read out contrast (C) and the spin coherence time $T_{2}$ generally quoted in naturally abundant isotope SiC.

In this study we will consider that the Purcell enhancement $F_{p}$ is generally reduced by the DW factor (χ) and the resonator-color center spatial overlap, so that the radiative emission enhancement can be calculated as [47]

(2) $$RE=F_{p}\cdot \chi \cdot \frac{E}{E_{max}}\cdot \cos\phi ,$$

where $E_{max}$ is the maximum electric field of the resonator mode, *E* is the electric field intensity at the color center, and ϕ is the angle between the orientation of color center dipole and the electric field [47]. We will determine the CPR=RE·CE achievable values for optimised diameter of coupled the SiC nanopillar and the Ag nanoantenna with the dipole source located at a fixed distance of 20 nm away from the bottom surface of the Ag cylinder, for the color centres properties as in Table 1.

We would like to point out that a detailed study on the point dipole emission coupling to various types of antennas (cylindrical, cuboid, bow-tie and cross) has already been presented in a previous work [48]. The CE for the cylindrical antennas is known to be among the best for the coupling of near-surface single color-center’s emission in bulk substrate. Here, since the emitter is based on a SiC pillar, for a feasible fabrication we are considering the SiC pillar diameter and the Ag antenna diameter to be the same. The coupled SiC pillar/Ag antenna scheme is optimised for the maximum collected photons or power collection from the structure. For dipole emission in a resonator, total CE mainly depends on the antenna dimensions [48]. Therefore, to observe the maximum CE, the Ag antenna’s diameter should be optimised independent of the SiC pillar’s diameter. However, the fabrication of such a structure with different diameter for the SiC pillar and the Ag antenna will be quite complicated.

## 3. Materials and Methods

The electrodynamics calculations are carried out using the commercial finite-element method based (FEM) Comsol Multiphysics Radio Frequency (RF) module. The second-order scattering boundary conditions are used for the outer boundaries of the computational domain. Within the calculation domain, the minimum mesh size is 0.5 nm and the maximum mesh size is λ/7. The Ag and SiC permittivity values are taken from [49,50] respectively.

### 3.1. Purcell Factor Calculations

Spontaneous emission is purely a quantum mechanical phenomenon. However, the relative spontaneous emission rates of an emitter scaled to a reference system are exactly the same under both the classical and quantum pictures [51]. In the classical picture, the influence of the local electromagnetic environment on the spontaneous emission of a radiating dipole is governed by the the local optical density of states (LDOS) [51]. The LDOS, ρ(ω,r) is defined as [52]

(3) $$\rho \left(\omega ,r\right)=\sum_{k,\sigma }{\left|\hat{d}\cdot E_{k,\sigma }\left(r\right)\right|}^{2}\delta \left(\omega -\omega_{k,\sigma }\right)$$

here, $\hat{d}$ is the unit vector along the direction of the transition dipole moment and ω is the transition frequency. The summation is over all available wave-vectors (k) and polarizations (σ). **E** is the total electric field at the source dipole emitter’s position. It is the superposition of the fields directly radiated by the dipole source with those scattered or reflected back from the surroundings. The LDOS is controlled the the amplitude of the electric field at the source’s position, the orientation of the electric field vector relative to the transition dipole moment vector and the total optical states available for the dipole source to couple its emitted radiation. Tuning any of these parameter, will tune the LDOS and hence the spontaneous emission rate of a dipole emitter.

In these calculations, the color-center based dipole-emitter is taken as a point current source, harmonically driven at the emission frequency, $\omega_{0}=2\pi \nu_{0}$ ($\nu_{0}=c/\lambda$) and the current density, $J=J\delta (\omega -\omega_{0})$ [51]. The total power radiated by the source is measured over a closed surface enclosing the point dipole-emitter. The Purcell factor, $F_{p}$ is calculated as $F_{p}=\frac{\gamma }{\gamma_{bulk}}=\frac{P}{P_{bulk}}$ [51].

### 3.2. Collection Efficiency Calculations

The total collection efficiency (CE) in a fluorescence measurement is defined as the fraction of the total radiated power which is collected by the collection objective lens. The total power being collected by an objective lens depends on the projected solid-angle its surface makes relative to the dipole emitter [5]. In our calculations, the CE is calculated by scaling the time-averaged power collected over the surface of a circular disc whose radius corresponds to the numerical aperture (NA) of the collection objective with the total power collected over a closed surface enclosing the point dipole-emitter. For the calculations, the NA is considered to be 0.9 which corresponds to a solid-angle of $64.15^{\circ }$). To avoid significant contributions resulting from the evanescent fields around the top surface of the metallic Ag pillar, the circular disc is kept at a distance of more than $\frac{3}{4}\lambda$ above the Ag top surface [52]. To test our calculations, we verified our calculated CE values against the analytically obtained CE values for the case of dipole radiation in the vicinity of a planar dielectric interface [53].

## 4. Results

**Figure 2:**
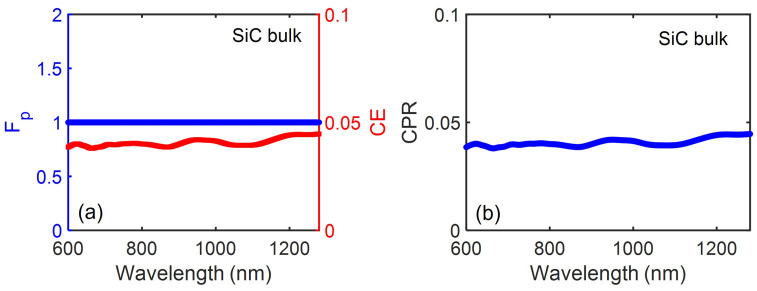
(**a**) The modified Purcell factor, $F_{p}$ and collection efficiency, CE and (**b**) the corresponding CPR performance for a point dipole emitter in bulk SiC as a function of the emission wavelength.

To develop a complete understanding of the role of the SiC pillar/Ag antenna scheme in tuning the spontaneous emission process of a SiC based color center, we first studied the emission performance of a point dipole emitter in the reference bulk SiC substrate. Here, the dipole is oriented perpendicular the c-axis (along the horizontal plane) and is positioned at a distance of 685 nm below the top surface. These results are shown in Figure 2. The total internal reflection (TIR) occurring at the bulk SiC/air interface for the embedded dipole’s emission, drastically limits the dipole emitter’s radiation coming out from the high refractive indexed SiC substrate. CE was therefore observed to be below 5% with the CPR being less than 0.05 throughout the wide emission spectrum ranging from 600 nm to 1280 nm. For a dipole oriented along the c-axis (along the vertical plane), since the dipole’s radiation is directed away from the dipole’s axis, the angle of incidence of the dipole’s radiation at the bulk/air interface will increase, thereby increasing the TIR fraction substantially. The CE was observed to be reduced further to about 0.5% with a CPR of 0.005 for a dipole source oriented along the c-axis [25].

**Figure 3:**
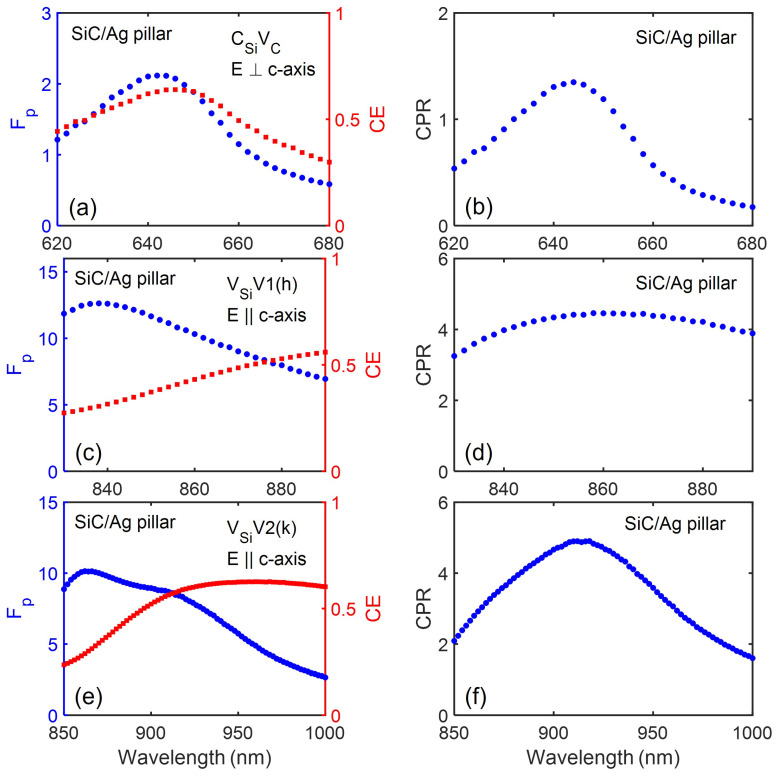
The modified Purcell factor, $F_{p}$, (**a**,**c**,**e**) and collection efficiency, CE; the corresponding CPR performance for various point defect emitters in SiC pillar (**b**,**d**,**f**). The specified color centres dipole is located 20 nm below the Ag cylinder surface with the given polarisations.

**Figure 4:**
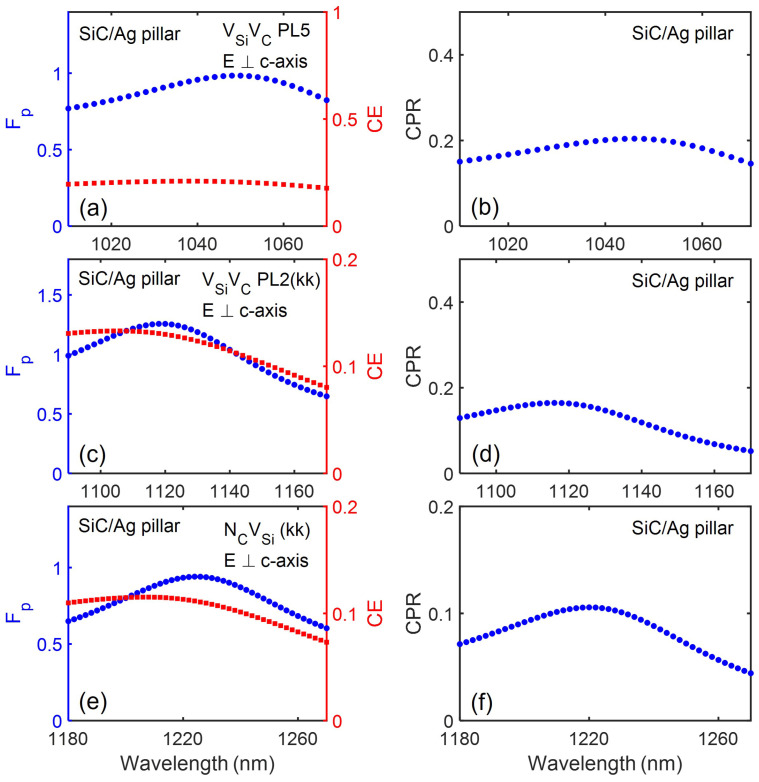
The modified Purcell factor, $F_{p}$ and collection efficiency, CE (**a**,**c**,**e**); the corresponding CPR performance for various point defect emitters in SiC pillar (**b**,**d**,**f**). The specified color centres dipole is located 20 nm below the Ag cylinder surface with the given polarisation.

Figure 3 and Figure 4 show the spectral emission performance of various color centres in 4H-SiC when placed in the SiC/Ag pillar antenna-resonator.

In this study, the color center is considered to be implanted close of the top surface. A detailed study on the influence of the color-center position within the SiC pillar is presented in a previous study [25]. Here, the study is focused on evaluating the complete spectral performance of the coupled SiC pillar/Ag antenna scheme for spontaneous emission enhancement of the wide range of single color centers observed in SiC substrate. Throughout this study, the color-centre based dipole emitter is radiating at a separation, d=20 nm below the SiC pillar’s top surface. For all the studied individual color centers, the SiC and Ag pillar’s diameters, *D* (both diameters being the same) and the Ag pillar’s height, $h_{1}$ are optimized independently to achieve maximum power collection from the dipole emitter. The height of the SiC pillar, $h_{2}$ is kept fixed in this study. Table 2 lists these parameters for the various studied color centers.

For emitters with dipole orientation parallel to the c-axis, $F_{p}$ enhancement of more than a factor of 10 can be observed for emission around 860 nm and 917 nm. This is close to the 7–8 times enhancement observed for the case of N${}_{C}V_{\mathrm{Si}}$ centers in SiC/Ag pillar antenna-resonator emitting at a much higher wavelength of 1310 nm [25]. For emitters with dipole orientation perpendicular to the c-axis, a reduction in the $F_{p}$ value ($F_{p}\leq 1$) due to quenching of the emission by the metallic Ag pillar is observed [54]. The CE value is observed to gradually decrease with an increase in the emission wavelength. For emission around 649 nm, the CE value was observed to be higher than 0.5 and it gradually reduced to a low value of about 0.1 for emission around 1223 nm. With an increase in the emission wavelength, the dimensions of the metallic Ag antenna increases. This will lead to higher absorption losses, thereby limiting the observed CE value.

**Figure 5:**
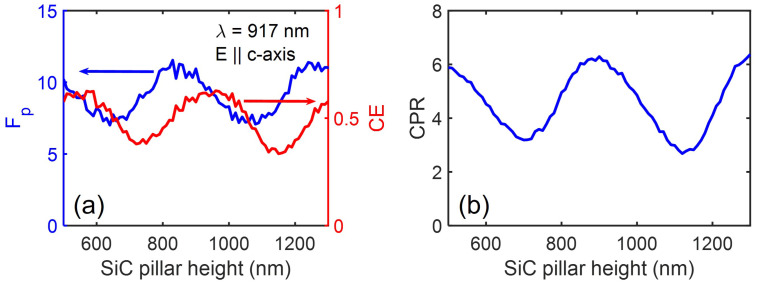
(**a**) The modified Purcell factor, $F_{p}$ and collection efficiency, CE; (**b**) the corresponding CPR performance as a function of the SiC pillar’s height variation for an embedded point defect emitters in SiC pillar emitting at 917 nm. The specified color centres dipole is located 20 nm below the Ag cylinder surface with polarisation along the c-axis.

We now studied the influence of the SiC pillar’s height, $h_{2}$, variation on the emission performance of the embedded color centres in 4H-SiC. These results are shown in Figure 5 for emission wavelength of 917 nm with the dipole orientation along the c-axis. The emission performance ($F_{p}$, CE and CPR) shows an oscillatory behaviour with the variation of the SiC pillar’s height. The $F_{p}$, CE and CPR oscillates between the maximum values 12, 0.6 and 6 and the minimum values 7, 0.35 and 3.5, respectively. The CPR is showing successive maxima for SiC pillar’s height variation of λ/2 with peaks corresponding to SiC pillar’s height of ∼460 nm, 920 nm and so on. The SiC cylinder is therefore acting as a lossy Fabry–Perot cavity along its length. For emission at 1223 nm with the dipole orientation perpendicular to the c-axis, the CPR oscillates between the maximum value 0.12 and the minimum value 0.08, respectively.

**Figure 6:**
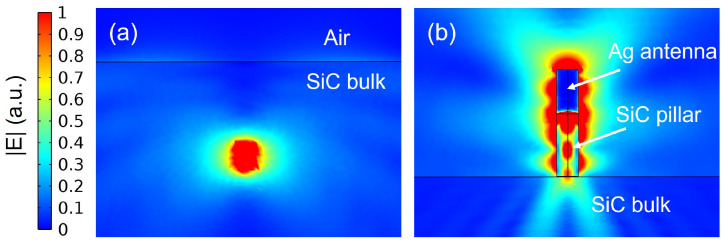
The 2-D normalised electric field pattern for a point dipole emission in (**a**) bulk SiC and (**b**) coupled SiC pillar/Ag antenna scheme optimized at 917 nm.

To develop an understanding on how the coupled SiC pillar/Ag antenna scheme is directing the dipole-emitter’s radiation pattern, Figure 6 shows the 2-D normalised electric field pattern for a vertically oriented (along the c-axis) point dipole emission at 917 nm in bulk SiC and SiC/Ag pillar antenna-resonator. The role of the Ag antenna placed on top of the SiC pillar in directing a substantial fraction of the dipole radiated fields along the vertically top direction can be observed here. This will lead to higher CE values. In both the cases (bulk SiC and SiC/Ag pillar antenna), it can be seen that the amplitudes of the electric fields at the position of the dipole source are of the same order. This limits the total LDOS and hence the Purcell enhancement as observed for the SiC/Ag pillar antenna-resonator.

**Figure 7:**
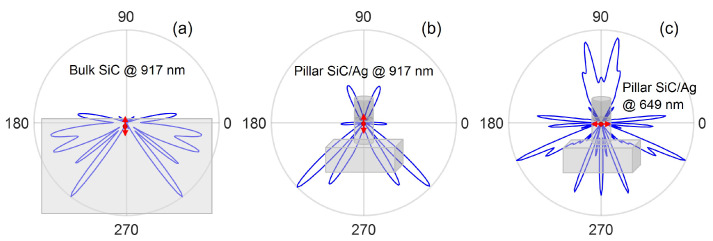
Far-field 2-D radiation patterns for a point dipole emission in (**a**) bulk SiC at 917 nm and SiC pillar at (**b**) 917 nm and (**c**) 649 nm, respectively.

The 2D far-field radiation profile for point dipole emission in bulk SiC and SiC/Ag pillar antenna-resonator is shown in Figure 7. For dipole emission in bulk SiC (Figure 7a), a very small fraction of the power comes out from the top surface (CE ∼ 0.5% for the dipole oriented perpendicular to the SiC-air interface). The use of the SiC/Ag pillar antenna-resonator scheme is observed to enhance the CE significantly. A CE of more than 50% is observed for a dipole oriented along the horizontal plane with emission at 649 nm (Figure 7c).

## 5. Conclusions

The emission performance of various known point defect centers in 4H-SiC coupled to metal-dielectric pillar antenna-resonator was studied in terms of $F_{p}$, CE and CPR. In bulk SiC, the emission performance is drastically reduced due to a very small CE due to substantial amount of total internal reflection at the SiC–air interface. In the SiC/Ag pillar antenna-resonator, the emission performance was found to be improved substantially with a high value of CE reaching around 60% for V1 and V2 lines and the A${}_{1}$ line of the $C_{\mathrm{Si}}V_{C}$. For dipoles oriented along the c-axis of the pillar such as $V_{\mathrm{Si}}$ V1 and V2, the $F_{p}$ was also found to be enhanced by an order of magnitude, leading to a relatively high value of CPR ∼5. For dipoles oriented perpendicular to the c-axis of the pillar such as $V_{\mathrm{Si}}V_{C}$ (PL2 and PL5) and $N_{\mathrm{Si}}V_{C}$, the $F_{p}$ was observed to be somewhat reduced to values ∼1. For these dipole, the CE was observed to gradually reduce with an increase in the emission wavelength due to larger metallic absorption losses occurring in the Ag antenna. The emission performance was observed to have an oscillatory response with the variation in the host SiC pillar’s height. The best CPR performance was observed for SiC pillars with heights in multiples of λ/2 with the maximum CPR value of 6. Overall, for the wide range of studied 4H-SiC colour centers, the CPR value was observed to vary within a range of 0.1 to 6. This is a significant enhancement from the very low CPR values of 0.005 (for dipoles along the c-axis) to 0.04 (for dipoles perpendicular the c-axis) for emission in bulk SiC. Based on this analysis this nano-antenna dielectric resonators is best suited to enhance the V1 and V2 lines of the $V_{\mathrm{Si}}$ and the A1 line of the $C_{\mathrm{Si}}V_{C}$.

## Figures and Tables

**Table 1:** Color centres in 4H-SiC zero phonon line (ZPL), the quantum efficiency (QE), and their optical and spin properties relevant for quantum technologies. Other relevant properties for the aforementioned applications such as the optical transition fluorescence lifetime ($\tau_{fl}$), optical spin-read out contrast (C), spin coherence time ($T_{2}$) are also listed. n.m. stands for not measured and n.a. for not available, s.f. stacking faults. The gyromagnetic ratio $g_{e}$ = 2.0028 for the $V_{\mathrm{Si}}$ [45].

Color Centre	ZPL	QE	$\tau_{\mathrm{fl}}$	χ	C	$T_{2}$
	(nm)	(%)	(ns)	(%)	(%)	(μs)
$C_{\mathrm{Si}}V_{C}$ [28]	649	70	1.2	2.3	n.a.	n.a.
E ⊥ c-axis	A${}_{1}$ (hh)			(calc.) [46]		
$V_{\mathrm{Si}}$	917	30	6.2	9	0.4–1.8	80
E ‖ c-axis	V2 (k)	[34]				[32]
$V_{\mathrm{Si}}$	862	n.m.	5.5	40	0.05–100	600
E ‖ c-axis	V1 (h)		[35]			[35]
$V_{\mathrm{Si}}$	858	n.m.	5.6	19	n.m.	n.m.
E ⊥ c-axis	V1’ (h)		[35]			
$V_{\mathrm{Si}}V_{C}$	1131	n.m.	14	5.3	5–6	1200 [39]
E ⊥ c-axis	PL2(kk)		[40]			
$V_{\mathrm{Si}}V_{C}$	1042	-	-	-	30	23
E ⊥c-axis	PL5 (s.f.)					[42]
$V_{\mathrm{Si}}V_{C}$	1038		-	-	23	23
E ‖c-axis [43]	PL6 (s.f.)					[42]
N${}_{C}V_{\mathrm{Si}}$	1223	98	2.7	-	<0.05	17
E ⊥c-axis [22]	(kk)	[22]				[20,21]
V${}^{4+}$ [44]	1279	2	167	25	-	-
	(h or α)					
V${}^{4+}$ [44]	1335	-	45	50	-	0.5
	(k or β)					

**Table 2:** The optimised parameters for the various studied color centres in 4H-SiC: the SiC/Ag cylinder diameter and Ag antenna height.

Color Centre	ZPL	Ag/SiC Diam.	Ag Height	SiC Height
	(nm)	(D) (nm)	(h${}_{1}$) (nm)	(h${}_{2}$) (nm)
$C_{\mathrm{Si}}V_{C}$	649	320	665	1000
E ⊥ c-axis	A${}_{1}$ (hh)			
$V_{\mathrm{Si}}$	917	370	705	1000
E ‖ c-axis	V2 (k)			
$V_{\mathrm{Si}}$	862	350	650	1000
E ‖ c-axis	V1 (h)			
$V_{\mathrm{Si}}V_{C}$	1131	684	1120	1100
E ⊥ c-axis	PL2(kk)			
$V_{\mathrm{Si}}V_{C}$	1042	630	1040	1100
E ⊥c-axis	PL5 (s.f.)			
N${}_{C}V_{\mathrm{Si}}$	1223	760	1200	1200
E ⊥c-axis	(kk)			

## Data Availability

Data are available upon request from the authors.
